# Reliability and construct validity of the Participation in Life Activities Scale for children and adolescents with asthma: an instrument evaluation study

**DOI:** 10.1186/1477-7525-6-43

**Published:** 2008-06-04

**Authors:** Eileen K Kintner, Alla Sikorskii

**Affiliations:** 1 Michigan State University College of Nursing, East Lansing, MI, USA

## Abstract

**Background:**

The purpose of this study was to evaluate the reliability and construct validity of the Participation in Life Activities Scale, an instrument designed to measure older school-age child and early adolescent level of involvement in chosen pursuits.

**Methods:**

A cross-sectional design was used. The convenience sample consisted of 313 school-age children and early adolescents with asthma, ages 9–15 years. The self-report summative scale of interest is a 3-indicator survey. Higher scores are reflective of higher levels of participation. Internal consistency reliability and construct validity for the entire sample and sub groups of the sample were evaluated.

**Results:**

The instrument was deemed sound for the entire sample as well as sub groups based on sex, race, age, socioeconomic status, and severity of illness. Cronbach's alpha coefficient for internal consistency reliability for the entire sample was .74. Exploratory factor analysis indicated a single component solution (loadings .79–.85) accounting for 66% of the explained variance. Construct validity was established by testing the posed relationship between participation in life activities scores and severity of illness. Confirmatory factor analysis revealed a good fit between the data and specified model, χ^2^(10, *n *= 302) = 8.074, *p *= .62.

**Conclusion:**

This instrument could be used (a) in clinical settings to diagnose restricted participation in desired activities, guide decision-making about treatment plans to increase participation, and motivate behavioral change in the management of asthma; and (b) in research settings to explore factors influencing and consequences of restricted and unrestricted participation, and as an outcome measure to evaluate the effectiveness of programs designed to foster child and early adolescent management of asthma.

## Background

Adolescents interviewed during a phenomenology study shared that participation in self-selected life activities was their primary motivator for behavioral change in coming to accept asthma as a chronic condition requiring ongoing monitoring and management [1]. Findings of the qualitative study defined participation in life activities as one's level of unrestricted involvement in chosen pursuits such as sports, clubs, interests, and hobbies [1-4]. The Participation in Life Activities Scale (PLA) [5] was developed to measure the concept.

Indicators for this concept were isolated from themes and statements extracted from the interviews to distinguish participation in life activities from other related concepts such as quality of life outcomes and domains of activity limitations. The indicators addressed levels of (a) planning for participation in activities due to asthma, (b) interference with participation, and (c) prevention from participation. The PLA uses five activities for each indicator. Activities are allowed to change over time as children grow and develop because the activities are not as important as the level of planning or restriction from participation believed to motivate changes in self management.

A copy of the instrument is provided elsewhere [5] along with details about the scale's development including theoretical foundations; evaluation of face and content validity; and preliminary cross-group comparison of scores based on sex, race, socioeconomic groupings, and severity of illness ratings. Face and content validity of the qualitatively-derived and theoretically-based instrument were determined to be highly acceptable and relevant by lay and expert reviewers. The PLA was deemed appropriate, useful, and applicable for both males and females ranging in age from 9–15 years of African American (Black) and Non-Hispanic Caucasian American (White) origins and from varying socioeconomic backgrounds [5].

### Purpose

The purpose of this paper is to report on internal consistency reliability and construct validity of the Participation in Life Activities Scale (PLA) for older school-age children and early adolescents diagnosed with asthma. After reliability and validity are demonstrated, healthcare professionals and researchers will be able to diagnose restricted participation in desired activities, explore factors influencing participation, examine consequences of participation, and evaluate efficacy of interventions to increase participation. Older school-age children and early adolescents will hereafter be referred to as children.

### Review of demographic and condition severity/control considerations

When examining reliability and validity of the PLA, sex/gender, race, age, and socioeconomic status should be considered. Preliminary cross-group comparisons indicated significant difference in PLA scores between males and females, and lowest to highest socioeconomic groups [5]. More research is needed to explore similarities and differences in scores based on race between Black and White Americans.

#### Demographic considerations

Asthma-related health statistics indicate that children, ages 9–14 years, experience increase morbidity and mortality over all other age groups, as do females over males, members of Black over White American groups, and families of lower socioeconomic groups over middle or upper groups [6-10]. Therefore, health disparities based on sex/gender, race, age, and socioeconomic status, which may influence participation in life activities, must be taken into account when selecting, developing, and evaluating instruments used for assessment purposes.

#### Condition severity/control considerations

Severity of asthma and level of symptom control greatly impact level of participation in life activities [2-4] and overall quality of life [11]. Due to stimuli exposure and subsequent exacerbation of symptoms, children with asthma are often restricted from participating in normal everyday activities such as laughing with friends, swimming in chlorinated pools, riding horses, playing with pets, going to camp, eating certain foods, being indoor or outdoor, exercising, and sleeping [11-17]. Without the use of effective medical treatments and management techniques to control symptoms, children may be limited by the severity of their condition.

In preparation for instrument testing, severity of illness or level of symptom control was hypothesized to be negatively associated with participation in life activities. Severity of illness is defined as the relative difficulty, effort, or struggle involved in controlling symptoms of one's chronic condition. This concept was operationalized through the use of the Severity of Illness Rating Scheme [18].

In summary, the qualitatively-derived PLA scale, designed to measure the adolescent identified outcome variable in the process of coming to accept asthma as a chronic condition is consistent with expert panel national guidelines and outcome criteria for treatment and management of childhood asthma [19]. Health statistics warrant examining psychometric properties of sub groups based on sex/gender, race, age, socioeconomic status, and severity of illness. Literature supports the hypothesis that states severity of illness is negatively associated with participation in life activities.

## Methods

### Design

A cross-sectional design was used. Data from three studies were combined to evaluate psychometric properties. The studies were in full compliance with the Helsinki Declaration and Health Insurance Portability and Accountability Act (HIPAA) requirements. Prior to data collection, human subjects' approvals were obtained through the University of Arizona Health Sciences Center Review Board for subjects recruited primarily in Arizona (1995–1996), the University of Michigan Health Sciences Institutional Review Board for subjects recruited in Michigan and Ohio (2001–2004), and Michigan State University Biomedical Institutional Review Board for subjects recruited in south-central Michigan (2005–2007). For all studies, written consent from a parent or legal guardian and assent from the child was obtained prior to data collection.

### Power analysis

In determining sample size for psychometric testing, the number of items contained in the target instrument, sensitivity of other instruments being used, and data analysis techniques were considered. Based on equations provided by Kim [20], 80% power for rejection of the proposed confirmatory factor model using a Root-Mean-Square Error of Approximation [21] of .05 and degrees of freedom required a minimum sample size of 214 subjects.

### Sample

The convenience sample consisted of 313 children, ages 9–15 years (*M *= 11.53, *SD *= 1.62), who lived in northern lower, south-eastern, and south-central Michigan (*n *= 14, 4.5%, *n *= 35, 11.1% and *n *= 153, 48.9%); southern Arizona (*n *= 80, 25.6%); north-western Ohio (*n *= 27, 8.6%); and central Oklahoma (*n *= 4, 1.3%).

In addition to 77 (24.6%) Black and 180 (57.5%) White American participants, the following racial/ethnic groups were represented: Asian American (*n *= 1), Latino/Mexican/Hispanic American (*n *= 20), Middle Eastern American (*n *= 1), Mixed (*n *= 16), Native American (*n *= 8), Other (*n *= 2), and Pacific Islander (*n *= 1) with other families (*n *= 7) not reporting.

### Return rate

For the first two studies, of the 318 paper-and-pencil packets mailed, 219 (69%) were returned. For the third study, of the 125 families approached, 107 were recruited, and 94 (88%) were enrolled and completed the surveys.

### Data collection

Data were collected from children diagnosed with asthma, ages 9–15 years, who were able to read and understand English. Flyers advertising the studies were offered to families through physicians' offices and schools. Families interested in learning about the study contacted the PI. After being informed of the purpose and nature of the study, requirements and responsibilities of subjects, and risks and benefits, families agreeing to participate in the first two studies were mailed a questionnaire packet. For the third study, home visits were scheduled for data to be collected using laptop computers. All items were entered into a user-friendly data entry system. The system was then audio-linked so that when participants clicked on icons, items and response options were read aloud in English.

Students completed the PLA. Parents/caregivers completed the General Health History Survey [2-4], and the Severity of Illness Rating Scheme [18]. Families that returned completed questionnaires were offered an award of $5 for the first study, $10 for the second study, and $30 for the third study. For the first two studies, healthcare providers who recruited eligible subjects were paid $5 per family that returned completed questionnaires. For the third study, school nurses were reimbursed for the time they served as recruiters on the study.

### Instruments

**The *Participation in Life Activities *(PLA) scale **is a 3-indicator scale designed to measure level of unrestricted involvement in chosen life activities [2-5]. Subjects are asked to list their most favorite activities then answer three activity-questions about each of them. Indicators measured by the activity-specific questions are cited below:

1. How much thinking about asthma is required when planning for participation in your favorite activities?

2. How much does asthma interfere with participation in your favorite activities?

3. How much does asthma prevent participation in your favorite activities?

#### Activity selection

Subjects may self-select anywhere from 1 to 5 or more favorite activities. A list of activities categorized under fun things to do as well as clubs, crafts, and sports is provided. Subjects may choose from the list or select activities not included on the list. Because participation in activities was the prime motivator for behavioral change by adolescents who were accepting of their asthma, having subjects select their own activities is imperative. When children are not vested in activities, then little will motivate the non-normative behavioral changes necessary for managing a chronic condition. Although five spaces for activities are provided on the survey form, the numbers and types of activities are not as important as their motivating influences. Numbers and types of activities must also be allowed to vary as children increase in complexity, differentiation, and specialization; while increasing in hierarchical integration and organization [5]. The activities serve as anchors for responding to three questions:

1. Do you need to think about asthma when planning to participate in this activity?

2. Does your asthma interfere with your participation in this activity?

3. Does your asthma ever keep you from participating in this activity?

#### Scoring

**Figure 1:**
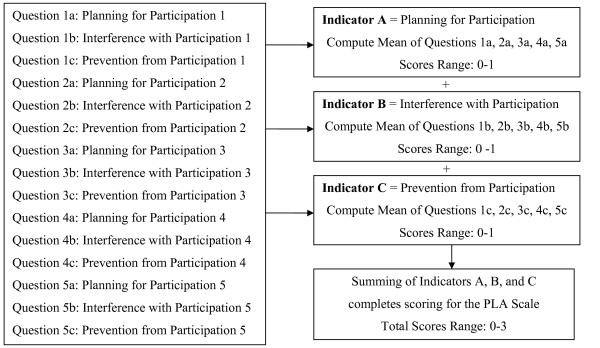
Scoring of the Participation in Life Activities Scale is completed by computing the mean scores for each of the three indicators before summing the indicator scores.

Subjects receive 0 points for answering "YES" and 1 point for answering "NO" to each of the activity-specific questions. Figure 1 offers an overview of scoring. Mean scores are computed for each of the three indicators: planning for participation, interference with participation, and prevention from participation. Indicator scores can range from 0–1 with higher scores reflective of less planning, less interference, and less prevention or rather increased participation. Since each indicator score is the mean across five activities, the variables are considered approximately continuous. Computing the sum across all three indicators completes scoring for the total scale. Total scale scores have potential to range from 0–3 with higher scores indicating greater participation in life activities.

Actual scores for all three indicators were in intervals ranging from 0 to 1. The mean score of planning was .482 (*SD *= .334), interference was .586 (*SD *= .320), and prevention was .749 (*SD *= .313). Total PLA scores ranged from 0–3 (*M *= 1.816, *SD *= .785).

***General Health History Survey ***is a 36-item survey completed by parents designed to collect demographic and disease-related information [2-4]. Demographic information reported here relates to sex/gender, race, age, and socioeconomic status. Socioeconomic status was computed using the Nam-Powers Socioeconomic Index Scores (SEIS) by averaging parents' occupation and education scores, and family income score [22]. SEIS scores have the potential to range from 0–100. The SEIS has demonstrated an extremely high degree of stability in status scores with correlation coefficients of .97 over 10 years, and .91 over 20 years [23]. The socioeconomic index scores were grouped into two categories representing lower to low middle (3–69) and upper middle to upper (70–99) levels. Disease specific information reported here includes a subjective measure of severity. Parents were asked to classify if their child's asthma was mild, moderate, or severe.

***Severity of Illness Rating Scheme ***is a multidimensional, 4-item instrument measuring severity of asthma tapping both pathophysiological aspects and responses to the condition. The scale uses amounts and types of medication necessary to control symptoms in combination with frequency of sleep and activity disturbance to form a summative composite score. Scores range from 4–12 with higher scores indicating higher severity. Concurrent validity was supported with significant correlations with parents' perceptions of their children's health status, school attendance records, and number of medical visits and hospitalizations. Scores for this sample ranged from 4–12 (*M *= 6.01, *SD *= 1.81). With item-to-total correlations of .39–.44, the standardized Cronbach's alpha reliability coefficient for internal consistency for the combined sample was lower than anticipated at .66, possibly due to the low item count.

### Data analysis

Descriptive statistics were computed for all measures.

**Internal consistency reliability **was assessed using Cronbach's alpha coefficient and item-to-total correlations. Because the PLA was developed for use in heterogeneous populations, reliability and construct validity assessments were conducted on the entire sample as well as sub groups based on sex, race, age grouping, family's socioeconomic status, and severity of illness.

#### Construct validity

Construct validity was evaluated using exploratory factor analyses of the three continuous PLA item indicators for the entire sample and sub groups. To obtain evidence of concurrent validity, the Pearson's correlation coefficient between PLA score and SIRS score was computed. Confirmatory factor analysis with 2 factors, PLA (defined by 3 continuous indicators), and SIRS (defined by 4 categorical/ordinal indicators) and a path from SIRS to PLA was carried out using weighted least squares method appropriate for ordinal categorical indicators [24]. Root Mean Square Error of Approximation (RMSEA) and Comparative Fit Index (CFI) were assessed [25]. In addition, chi-square test was used to evaluate model fit.

Internal consistency reliability analyses and exploratory factor analyses were conducted in SPSS for Windows 14.0.2 [26]. Confirmatory factor analysis was implemented using Mplus software [27].

## Results

### Internal consistency reliability

With strong corrected item-to-total correlations (*r *= .52–.63), the standardized Cronbach's alpha reliability coefficient for internal consistency of the Participation in Life Activity (PLA) Scale for this sample (*N *= 304) was .74. Tables 1, 2, 3, 4, 5, 6 and 7 present scale item/indicator summaries citing the number of subjects, corrected item-to-total correlations, and alpha-if-item-deleted for the combined sample as well as selected sub groups based on sex/gender, race, age, socioeconomic status, and severity of illness.

**Table 1:** Item Summary, Internal Consistency Reliability, and Results of Exploratory Factor Analysis for the Entire Sample (N = 304)

			Internal Consistence Reliability		Factor Analysis Principal Components		
	*M*	*SD*	Item to Total Correlation	Scale Alpha & if Item Deleted	KMO Sampling Adequacy	Rescaled Factor Loading (PC)	Rescaled Amount of Variance Explained
PLA	1.816	.785	-	.741	.670	-	65.8
Planning for	.482	.334	.52	.716	-	.797	-
Interference with	.586	.320	.63	.583	-	.846	-
Prevention from	.749	.313	.56	.662	-	.791	-

**Table 2:** Item Summary, Internal Consistency Reliability, and Results of Exploratory Factor Analysis for Females (n = 147) and Males (n = 157)

			Internal Consistence Reliability		Factor Analysis Principal Components		
	*M*	*SD*	Item to Total Correlation	Scale Alpha & if Item Deleted	KMO Sampling Adequacy	Rescaled Factor Loading (PC)	Rescaled Amount of Variance Explained
Females PLA	1.707	.817	-	.770	.691	-	68.4
Planning for	.478	.337	.58	.718	-	.824	-
Interference with	.551	.333	.65	.641	-	.858	-
Prevention from	.678	.317	.59	.707	-	.798	-
							
Males PLA	1.919	.742	-	.711	.653	-	63.2
Planning for	.486	.332	.46	.701	-	.796	-
Interference with	.618	.306	.59	.534	-	.826	-
Prevention from	.815	.295	.53	.615	-	.762	-

**Table 3:** Item Summary, Internal Consistency Reliability, and Results of Exploratory Factor Analysis for Black Americans (n = 69) and White Americans (n = 177)

			Internal Consistence Reliability		Factor Analysis Principal Components		
	*M*	*SD*	Item to Total Correlation	Scale Alpha & if Item Deleted	KMO Sampling Adequacy	Rescaled Factor Loading (PC)	Rescaled Amount of Variance Explained
Black PLA	1.737	.914	-	.823	.704	-	73.8
Planning for	.491	.362	.62	.814	-	.828	-
Interference with	.549	.336	.72	.716	-	.867	-
Prevention from	.696	.366	.70	.730	-	.883	-
							
White PLA	1.929	.716	-	.685	.646	-	60.7
Planning for	.495	.334	.49	.601	-	.851	-
Interference with	.636	.301	.57	.497	-	.818	-
Prevention from	.798	.277	.44	.658	-	.665	-

**Table 4:** Item Summary, Internal Consistency Reliability, and Results of Exploratory Factor Analysis for Students Ages 9–11 years (n = 162) and 12–15 years (n = 142)

			Internal Consistence Reliability		Factor Analysis Principal Components		
	*M*	*SD*	Item to Total Correlation	Scale Alpha & if Item Deleted	KMO Sampling Adequacy	Rescaled Factor Loading (PC)	Rescaled Amount of Variance Explained
Ages 9–11 PLA	1.767	.802	-	.768	.680	-	68.4
Planning for	.475	.326	.57	.726	-	.806	-
Interference with	.570	.320	.66	.616	-	.862	-
Prevention from	.722	.324	.58	.716	-	.810	-
							
Ages 12–15 PLA	1.872	.764	-	.708	.655	-	62.8
Planning for	.490	.344	.46	.699	-	.796	-
Interference with	.603	.320	.58	.542	-	.823	-
Prevention from	.779	.299	.54	.598	-	.759	-

**Table 5:** Item Summary, Internal Consistency Reliability, and Results of Exploratory Factor Analysis for Socioeconomic Scores reflective of Lower to Low middle (n = 170) and Upper middle to Upper (n = 133) levels

			Internal Consistence Reliability		Factor Analysis Principal Components		
	*M*	*SD*	Item to Total Correlation	Scale Alpha & if Item Deleted	KMO Sampling Adequacy	Rescaled Factor Loading (PC)	Rescaled Amount of Variance Explained
Lower (3–69) PLA	1.719	.816	-	.739	.657	-	65.8
Planning for	.439	.341	.473	.756	-	.747	-
Interference with	.560	.335	.605	.602	-	.841	-
Prevention from	.720	.331	.616	.589	-	.843	-
Upper (70–99) PLA	1.951	.718	-	.721	.633	-	65.2
Planning for	.541	.316	.554	.621	-	.852	-
Interference with	.621	.298	.640	.511	-	.865	-
Prevention from	.790	.282	.444	.743	-	.672	-

**Table 6:** Item Summary, Internal Consistency Reliability, and Results of Exploratory Factor Analysis for Parent Perception of Condition Severity as Mild (n = 137) and Moderate to Severe (n = 163)

			Internal Consistence Reliability		Factor Analysis Principal Components		
	*M*	*SD*	Item to Total Correlation	Scale Alpha & if Item Deleted	KMO Sampling Adequacy	Rescaled Factor Loading (PC)	Rescaled Amount of Variance Explained
Mild PLA	1.974	.772	-	.709	.663	-	62.6
Planning for	.538	.354	.481	.674	-	.824	-
Interference with	.631	.329	.573	.546	-	.824	-
Prevention from	.804	.288	.523	.619	-	.720	-
More Severe PLA	1.694	.777	-	.758	.672	-	67.5
Planning for	.441	.310	.527	.740	-	.762	-
Interference with	.553	.310	.655	.597	-	.855	-
Prevention from	.670	.328	.538	.681	-	.842	-

**Table 7:** Item Summary, Internal Consistency Reliability, and Results of Exploratory Factor Analysis for Severity of Illness Rating Scores (SIRS) reflective of Mild (n = 150) and Moderate to Severe (n = 153)

			Internal Consistence Reliability		Factor Analysis Principal Components		
	*M*	*SD*	Item to Total Correlation	Scale Alpha & if Item Deleted	KMO Sampling Adequacy	Rescaled Factor Loading (PC)	Rescaled Amount of Variance Explained
SIRS (4–5) PLA	1.989	.740	-	.689	.660	-	60.7
Planning for	.542	.349	.50	.598	-	.858	-
Interference with	.635	.317	.55	.529	-	.811	-
Prevention from	.812	.274	.47	.641	-	.654	-
SIRS (6–12) PLA	1.653	.794	-	.764	.658	-	68.0
Planning for	.424	.310	.50	.781	-	.728	-
Interference with	.541	.315	.68	.589	-	.871	-
Prevention from	.688	.338	.62	.663	-	.867	-

### Construct validity

#### Exploratory factor analysis

The principal components extraction method from the covariance matrix with no rotation was used for exploratory factor analyses. The eigenvalues and scree plot indicated a single component solution (loadings .80, .85, and .79 respectively) accounting for 66% of the explained variance. Tables 1, 2, 3, 4, 5, 6 and 7 present an overall scale summary citing Kaiser-Meyer-Olkin (KMO) Measures, component loadings, and percent of variance explained for the combined sample as well as selected sub groups based on sex/gender, race, age, socioeconomic status, and severity of illness.

#### Concurrent validity

Participation in life activities was negatively associated with severity of illness (*r *= -.24, *p *= .000).

#### Confirmatory factor analysis

**Figure 2:**
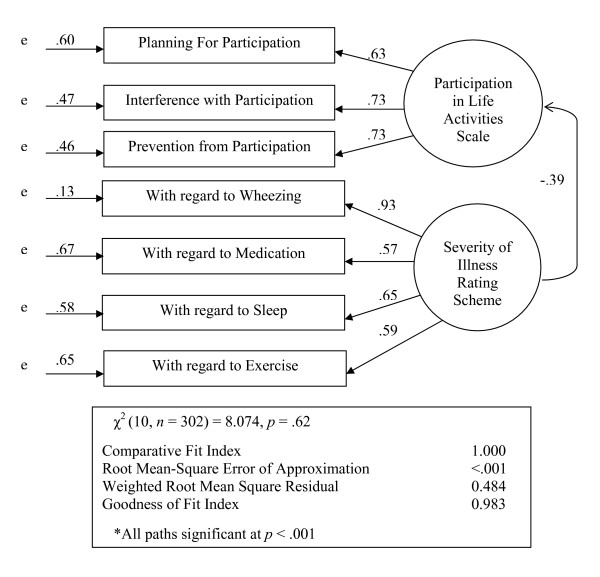
The Confirmatory Factor Model for Participation in Life Activity Scale and Severity of Illness Rating Scheme indicates goodness of fit between the data and the specified model.

The model estimation terminated normally and resulted in chi-square *p*-value of .62 for the test of model fit. The values of RMSEA < .001 and CFI of 1.00 were also indicative of good fit of the model. Factor loadings for each indicator were significant, and presented in Figure 2. The association between the two factors was significant as reflected by the standardized coefficient of -.39, *p *= < .001.

## Discussion

Psychometric evaluation of the qualitatively-derived and theory-guided PLA scale for children and adolescents with asthma demonstrated internal consistency reliability and construct validity based on exploratory and confirmatory factor analysis techniques for the combined sample as well as sub samples represented by sex/gender, race, age, socioeconomic status, and severity of illness. Preliminary cross-group comparisons in PLA mean scores indicated significant differences based on sex/gender and socioeconomic group. Background literature suggested that health disparities related to age, race, and severity of condition as well as sex and socioeconomic status could impact scores and should therefore be considered when evaluating the instrument.

Overall reliability for this newly developed, 3-indicator instrument was good. Corrected item-to-total corrections for the entire scale as well as various sub groups were more than adequate. Cronbach's alpha coefficient for internal consistency reliability was higher for younger than older children, females than males, Black than White Americans, and children with reported more severe conditions than those with less severe conditions, although not to a degree that would engender barriers for use.

Exploratory and confirmatory factor analyses revealed similar findings for construct validity with regard to component and factor loadings. Percent variance explained was higher for younger than older children, females than males, Black than White Americans, and children with reported more severe conditions than those with less severe conditions. Reliability and validity based on exploratory factor analysis did not appear to vary between groups based on socioeconomic status. Concurrent validity was supported by associations between participation in life activities and severity of illness with statistically significant correlation and path coefficients.

Psychometric and clinimetric evaluation using test-retest and pre- to post-test assessment techniques are needed to provide information about stability and change in scores over time. Having a parent or caregiver complete the same form would offer an indication of inter-rater reliability for equivalence. Comparisons of contrasting groups and investigations of associations with measures of related constructs could offer additional support for validity. For example, comparing scores for children classified with mild intermittent verses severe persistent disease, and comparing scores and psychometric evaluation of this instrument with the activity limitation and physical functioning items of the Pediatric Asthma Quality of Life Questionnaire (PAQLQ) [28] and Pediatric Quality of Life Inventory™ Generic Core Scales & Asthma Module (PedsQL™) [29] could be enlightening.

Adolescents with asthma identified unrestricted participation in life activities as their prime motivator for behavioral change in coming to accept asthma as a chronic condition requiring ongoing monitoring and management. Evidence indicates that for this target age group, support from healthcare professionals, parents, caregivers, and best friends fosters participation in life activities [2,3] and consequently, participation in life activities enriches psychosocial outcomes such as self-perception of athletic competence, physical appearance, social acceptance, and global self-worth, as well as perceived social support from classmates and schoolteachers [2,3].

Visual inspection of scores indicates that a large number of the subjects who were more restricted in participation selected physically challenging sports activities; whereas subjects who scored higher in participation tended to select more sedentary activities. From a lifespan development perspective, exploring whether or not experiences with asthma influence lifestyle choices is of particular interest. We considered coding favorite activities by level of exposure to potential stimuli as low, moderate, or high risk for exacerbation of symptoms. However, coding activities is complicated when individuals report that reading is a risk when exposed to news print or dusty books, going to the mall is a risk due to strong scents or perfumes, talking on the phone is a risk when laughing with friends, and sleeping at night is a risk without pillow and mattress protectors. Coding for levels of exposure to capture any one individual's specific stimuli and risk of symptom exacerbation would be challenging, especially when not all stimuli are known or clearly identified by children and their caregivers.

The PLA could be used by health care professionals in clinical settings to (a) diagnose restricted participation in favorite activities, (b) guide decision-making about treatment plans to increase participation, and (c) motivate behavioral change in the ongoing monitoring and management of asthma. The instrument could also be used (a) to explore factors influencing and consequences of restricted and unrestricted participation and (b) as an outcome measure to evaluate the effectiveness of theory-guided, research-based programs designed to foster school-age child and early adolescent self-management of their asthma.

## Conclusion

Findings of this study provide evidence of internal consistency reliability and construct validity of the PLA as a measure of one aspect of quality of life for children and adolescents with asthma.

## List of abbreviations

PLA: Participation in Life Activities Scale; HIPAA: Health Insurance Portability and Accountability Act; SEIS: Nam-Powers Socioeconomic Index Scores; KMO: Kaiser-Meyer-Olkin Measure; CFI: Comparative Fit Index; RMSEA: Root-Mean-Square Error of Approximation; PAQLQ: Pediatric Asthma Quality of Life Questionnaire; PedsQL™: Pediatric Quality of Life Inventory™ Generic Core Scales and Asthma Module.

## Competing interests

The authors declare that they have no competing interests.

## Authors' contributions

EK conceived of the study, served as Principal Investigator overseeing all aspects of all three studies conducted to obtain data, and performed the statistical analyses using SPSS for Windows to obtain descriptive data as well as internal consistency reliability and exploratory factor analysis estimates. AS performed the statistical analysis using Mplus to evaluate fit of the data to the hypothesized model. Both authors read and approved the final manuscript. The authors are solely responsible for the content contained in this article.
